# Predicted Input of Uncultured Fungal Symbionts to a Lichen Symbiosis from Metagenome-Assembled Genomes

**DOI:** 10.1093/gbe/evab047

**Published:** 2021-03-09

**Authors:** Gulnara Tagirdzhanova, Paul Saary, Jeffrey P Tingley, David Díaz-Escandón, D Wade Abbott, Robert D Finn, Toby Spribille

**Affiliations:** 1 Department of Biological Sciences CW405, University of Alberta, Edmonton, Alberta, Canada; 2 European Molecular Biology Laboratory, European Bioinformatics Institute (EMBL-EBI), Wellcome Trust Genome Campus, Hinxton, Cambridge, United Kingdom; 3 Agriculture and Agri-Food Canada, Lethbridge Research and Development Centre, Lethbridge, Alberta, Canada

**Keywords:** extracellular matrix, genome, metagenomics, Lecanoromycetes, mycoparasite, secretome, yeast

## Abstract

Basidiomycete yeasts have recently been reported as stably associated secondary fungal symbionts of many lichens, but their role in the symbiosis remains unknown. Attempts to sequence their genomes have been hampered both by the inability to culture them and their low abundance in the lichen thallus alongside two dominant eukaryotes (an ascomycete fungus and chlorophyte alga). Using the lichen *Alectoria sarmentosa*, we selectively dissolved the cortex layer in which secondary fungal symbionts are embedded to enrich yeast cell abundance and sequenced DNA from the resulting slurries as well as bulk lichen thallus. In addition to yielding a near-complete genome of the filamentous ascomycete using both methods, metagenomes from cortex slurries yielded a 36- to 84-fold increase in coverage and near-complete genomes for two basidiomycete species, members of the classes Cystobasidiomycetes and Tremellomycetes. The ascomycete possesses the largest gene repertoire of the three. It is enriched in proteases often associated with pathogenicity and harbors the majority of predicted secondary metabolite clusters. The basidiomycete genomes possess ∼35% fewer predicted genes than the ascomycete and have reduced secretomes even compared with close relatives, while exhibiting signs of nutrient limitation and scavenging. Furthermore, both basidiomycetes are enriched in genes coding for enzymes producing secreted acidic polysaccharides, representing a potential contribution to the shared extracellular matrix. All three fungi retain genes involved in dimorphic switching, despite the ascomycete not being known to possess a yeast stage. The basidiomycete genomes are an important new resource for exploration of lifestyle and function in fungal–fungal interactions in lichen symbioses.

**Significance**

Many lichen symbioses have been recently shown to contain low-abundance secondary fungal symbionts in the form of basidiomycete yeasts. Here, we present the first annotated genomes of the secondary fungal symbionts and compare them with the genomes of the dominant fungus of the symbiosis. Lichen yeast genomes are among the smallest 5% in fungi, but possess the machinery for secreted polysaccharide profiles and phosphate scavenging functions not found in the dominant fungal symbiont.

## Introduction

Culture-independent molecular methods have been a game changer for working with mutualistic symbioses, which are often recalcitrant to laboratory experimentation. Not only have such methods led to the discovery of previously unknown symbionts (e.g., Matsuura et al. 2018), they have also permitted us to explore their functional potential (e.g., Karimi et al. 2018). Lichen symbioses were long considered to consist entirely of a fungus and one or two photosynthesizing partners, usually a chlorophyte alga and/or a cyanobacterium, based on what could be interpreted with confidence using traditional microscopy. Despite evidence of additional associated microbes, including both bacteria and fungi, from culturing studies as early as the 1930s (Lenova and Blum 1983), it was only through shotgun sequencing that the stable and constant association of basidiomycete secondary fungal symbionts (SFSs) was discovered in lichen symbioses, especially those formed by members of the ascomycete family Parmeliaceae (Spribille et al. 2016; Tuovinen et al. 2019). These partners had not only evaded previous detection by culturing but also by amplicon sequencing with common primers (Spribille et al. 2016).

The inability to isolate SFSs has not only made them hard to detect, it has also left their relationship to the lichen difficult to test. In the lichen system in which they were first detected, the *Bryoria tortuosa* symbiosis, their abundance correlated positively with the visible production of the secondary metabolite vulpinic acid in the shared extracellular matrix between the core ascomycete symbiont and the yeasts. The close association with a secondary metabolite and the tight integration of yeasts into the extracellular matrix led us to hypothesize a role in contributing to secondary metabolism and/or in secreting polysaccharides into the extracellular matrix (Spribille et al. 2020). Perhaps not exclusive of these possibilities, other authors have suggested that SFSs may be parasites. The two main groups of SFSs, members of the basidiomycete orders Cyphobasidiales and Tremellales, were both known in lichens prior to their discovery as yeasts by their fertile, hyphal forms. These are rare but easier to spot than yeasts, in the form of gall-like protrusions on lichen thalli (Tuovinen et al. 2019, 2021). Their relationship to known mycoparasites has led others to suspect that they parasitize the core ascomycete symbiont (Millanes et al. 2016). That being said, we are not aware of any direct evidence of mycoparasitism, such as fungal–fungal haustoria, from lichen SFSs. We have, however, shown one of them (*Tremella*) to enmesh algal cells (Tuovinen et al. 2019).

It became evident in our original metatranscriptome study of SFSs that determining the nature of SFS interactions with the other members of the lichen system would not be trivial. In studies that have used raw mRNA extracts from whole lichens (Spribille et al. 2016, Tuovinen et al. 2019), the much lower cell abundance of the yeasts resulted in flow cell terminal space being swamped by cDNA from the more abundant core symbionts. The problem of core symbiont DNA driving down secondary symbiont coverage also manifests itself when sequencing metagenomic libraries. The ability to recover SFS reads declines as less flow cell space is dedicated to a whole library and appears to stand in direct relationship to declining coverage of the core symbionts. For instance, when the ascomycete symbiont is sequenced at 5× coverage, SFSs may not be detected at all in many cases (Lendemer 2019), even in lichen symbioses in which they are readily demonstrable at high frequency using endpoint PCR screening (Černajová and Škaloud 2019; Mark et al. 2020).

Even if deeper coverage is obtained, other hurdles have stood in the way of assembling complete and comparable eukaryotic genomes from metagenomic samples. Although microbial eukaryotes constitute a significant fraction of biodiversity and have recently gained more attention (Delmont et al. 2018; West et al. 2018), the recovery of high-quality metagenomic assembled eukaryotic genomes has been limited by the bioinformatic challenges presented by the larger genome size and complexity (e.g., repetitive regions and varied nucleotide composition). Solving these challenges could provide a powerful tool set to 1) interrogate the lichen system both for other stably associated symbionts, as well as 2) provide initial prognoses of the gene repertoires and potential complementarities of the genomes involved.

The present study had two specific goals. First, we set out to obtain high coverage genome assemblies for previously unobtainable low abundance partners from wild lichen material. We accomplished this by sequencing a metagenome both from whole lichen material as well as from slurry derived from dissolved lichen EPS. Second, we set out to predict the gene repertoires of the two SFSs associated in high frequency with the in vivo lichen and contrast them to the genome of the dominant fungal partner based entirely on metagenome-derived data sets. For this portion of the study, we focused on three aspects of their biology relevant for the lichen symbiosis: 1) potential contributions of the SFSs to the lichen symbiosis, including production of polysaccharide matrix and secondary metabolites, nutrient scavenging and lipid deposition; 2) trophic lifestyle of the SFSs and their relationship to the “core” fungus; and 3) the detection of potential signal for mutualistic versus antagonistic interactions between the fungi in the symbiosis. The findings run up against new limitations, but substantially extend our knowledge of the potential capabilities of the SFSs.

## Materials and Methods

### Sample Collection, Preparation, and Sequencing

For a whole lichen metagenome, we collected a thallus of *Alectoria sarmentosa* lichen on March 3, 2017 along the Lochsa River in Idaho County, Idaho, USA (46.56742°N, 114.63975°W). The sample was frozen at –80 °C and ground in a TissueLyser II (Qiagen, Hilden, Germany). We extracted DNA using DNeasy Plant Mini Kit (Qiagen) and prepared a metagenomic library using TruSeq DNA PCR-Free Low Throughput Library Prep Kit (Illumina, San Diego, CA). The library was sequenced at the Huntsman Cancer Center at University of Utah on an Illumina HiSeq 2500 using 125-bp paired-end reads.

We generated another metagenome enriched in low-abundance organisms embedded in the matrix of the cortical layer. For that we collected a healthy-looking thallus of *A. sarmentosa* in June 2018 at the edge of Wells Gray Provincial Park, British Columbia, Canada (51.76°N, 119.94°W). The lichen material was rinsed in water to remove contamination from the surface, put it in 200 ml of water and placed in a shaking incubator overnight at 60 °C. We centrifuged the resulting solution for 3 min at 30 × g to remove large pieces of lichen material. The remaining liquid was centrifuged for 7 min at 3,000 × g. We dried the resulting pellet overnight at 60 °C and extracted DNA as described above. A total of 10 ng of DNA was used for metagenomic library preparation. We prepared the library using NEBNext Ultra II DNA Library Prep Kit (New England BioLabs, Ipswich, MA). The library was sequenced at the BC Cancer Genome Sciences Centre on an Illumina HiSeq X using 150-bp paired-end reads.

### Metagenome Assembly and Binning

The libraries were filtered with the metaWRAP pipeline (v1.2, Uritskiy et al. 2018). Using bbmap (Bushnell 2014) within the READ_QC module, we aligned reads against hg38 to remove any human contamination. The remaining reads were then assembled into two individual metagenomes using metaSPAdes default settings (supplementary table S1, Supplementary Material online) (v3.13, Nurk et al. 2017). Individual assemblies were binned with CONCOCT within metaWRAP (Alneberg et al. 2014).

We used several tools to identify MAGs and assess their quality. First, we analyzed all bins using CheckM (v1.0.18, Parks et al. 2015), which gave taxonomic placement and quality estimation for prokaryotic MAGs. Then, we analyzed the quality of all bins using EukCC, which gave a first taxonomic assignment as well (Saary et al. 2020). To infer a taxonomic placement of all bins, we used models created by GeneMark-ES (v4.38, Lomsadze et al. 2014) for the almost complete bins of the same data set, to predict proteins in small and incomplete bins, which usually cannot be predicted with GeneMark-ES in the self-training mode. We then inferred taxonomic position by subsampling up to 200 proteins per bin and subsequently blasting them against the UniRef90 database (UniProt release: 2019_01) using Diamond’s BLASTp option (Buchfink et al. 2015). For each protein we considered the top 3 hits passing an e-value threshold of 1 × 10^−20^ and used a majority vote of 60% to assign the lowest common ancestor (LCA) per protein. Using the same majority vote, we assigned a LCA per bin as the sum of all sampled proteins. Additionally, we ran BUSCO (v4.0.1, Seppey et al. 2019) on all bins assigned to eukaryotes and, additionally, FGMP (Cissé and Stajich 2019) on all fungal bins. Basic statistics of all MAGs as well as the two metagenomic assemblies were calculated using QUAST (v4.5, Gurevich et al. 2013) using default settings. Median genome coverage was calculated using bowtie2 (v2.3.4.3, Langmead and Salzberg 2012) samtools (v1.8-1, Li et al. 2009), and a custom script (see details on https://github.com/metalichen/).

For further analysis, we took bins with >90% genome completeness according to at least one tool and <5% contamination. In cases where we had multiple highly similar genomes assigned to the same taxonomic group, we picked the genome with the highest completeness and for further analysis used only it; in case of lecanoromycete genomes we used the one isolated from the cortex-derived metagenome.

### Refining the Taxonomic Placement of the Genomes

We used protein predictions from the fungal MAGs to refine their taxonomic placement. We combined predicted proteomes (see the details on genome annotation below) with proteome data on 38 fungal species from published sources (supplementary table S11, Supplementary Material online). We used Orthofinder (v2.3.8, Emms and Kelly 2019) to identify single copy orthologs genes using Diamond (v0.9.29, Buchfink et al. 2015) all vs all pairwise similarity scores, and constructing a preliminary phylogeny using all shared orthologs genes using the STAG (Emms and Kelly 2018) algorithm to infer multi-copy gene trees within Orthofinder. We selected all single copy orthologs sequences resulting from Orthofinder, aligned them using MAFFT (v7.455, Katoh et al. 2002) and trimmed the low coverage sites using trimAl (v1.2rev59, Capella-Gutiérrez et al. 2009) under automatic settings. We constructed a consensus species tree concatenating all genes, using IQ-TREE (v2.0.2rc2, Nguyen et al. 2015) with a 1,000 repetitions thorough bootstrap and calculating partition evolutionary models per gene based on amino acids matrices. Then, we constructed gene trees for each single copy ortholog gene using the partition models calculated in IQTREE and run in RAxML (v8.2.12, Stamatakis 2014) a maximum likelihood analysis with 1,000 thorough bootstrap under a CAT model with an LG substitution matrix per gene (Le and Gascuel 2008), using CIPRES science gateway servers (Miller et al. 2010). The resulting gene trees were combined into a species tree using the coalescence-based method ASTRAL (v5.14.5, Zhang et al. 2018) calculating a local posterior probability for induced shared quartets based on 1,000 bootstrap trees per gene.

After narrowing taxonomic placement down to the class level, we used BlastN to extract sequences of ITS (internal transcribed spacer; rDNA) for Tremellomycetes and Cystobasidiomycetes from both metagenomic assemblies. We incorporated these into published sequences of their respective class from the literature; all sequences used in this analysis and their NCBI GenBank accession numbers are presented in supplementary table S12, Supplementary Material online. The taxon sampling was done partially following Spribille et al. (2016), Millanes et al. (2011) and Liu, Wang, et al. (2015). Each set of sequences were aligned using MAFFT (v7.271, Katoh et al. 2002) with the flags --genafpair --maxiterate 10000. The alignments were trimmed using trimAl (v1.4.rev15, Capella-Gutiérrez et al. 2009) to remove all sites with ≥90% missing data. We determined optimal nucleotide substitution model schemes using PartitionFinder (v2.1.1, Lanfear et al. 2012) with default config settings. Maximum likelihood phylogenetic analyses were performed using IQ-TREE (v1.6.12, Nguyen et al. 2015) with GTR+I+G substitution model and 50,000 rapid bootstrap replicates.

### PCR-Based Screening

To check whether the newly identified lineages are consistently present in *A. sarmentosa*, we performed PCR screening. We collected 32 thalli of *Alectoria* in three locations (supplementary table S7, Supplementary Material online). Each thallus was complemented with two specimens from the same tree: A lichen of a different species and a bark sample. DNA from the lichen material and pieces of bark was extracted as described above. Primers used for the screening are listed in supplementary table S13, Supplementary Material online. For screening *Cyphobasidium*, we used primers and PCR protocol described at Spribille et al. (2016). Screening *Tremella* was performed following Tuovinen et al. (2019). Amplification of *Granulicella* rpoB was done with annealing at 53 °C and 35 cycles. All PCR reactions were performed using KAPA 3G Plant PCR kit (Roche Sequencing Solutions, Pleasanton, CA). PCR products were cleaned prior to sequencing with Exonuclease I and Shrimp Alkaline Phosphatase (New England BioLabs, Ipswich, MA). Amplicons were sequenced by Psomagen Inc (Rockville, MD). We counted a lineage as present if the PCR reaction produced an assignable sequence. Taxonomy assignments of the sequences were verified either by searching them against the NCBI database (for low quality sequences) or by a phylogenetic analysis (supplementary table S14, Supplementary Material online). Produced sequences of mid and high quality were incorporated into published sequences of their respective groups (supplementary table S12, Supplementary Material online for *Cyphobasidium* and *Tremella*, supplementary table S15, Supplementary Material online for *Granulicella*). We produced phylogenetic trees in the way described above.

### Genome Annotation and Analyses

Functional annotation of the three fungal genomes isolated from the cortex-derived metagenome was performed using the Funannotate pipeline (v1.5.3, github.com/nextgenusfs/funannotate, last accessed February 8, 2021). The assemblies were cleaned to remove repetitive contigs, then sorted and repeat masked. The prepared assemblies were subjected to ab initio gene prediction using GeneMark-ES (v4.38, self-trained, Lomsadze et al. 2014) and AUGUSTUS (v3.3.2, trained using BUSCO2 gene models, Stanke et al. 2004). EVidenceModeler (v1.1.1, Haas et al. 2008) was used to create consensus gene models. Finally, the models shorter than 50 amino acids or identified as known transposons were excluded using BLASTp search.

Functional annotations were assigned to protein coding gene models using several pipelines: Output from InterProScan (v1.5.3, Jones et al. 2014) and Eggnog-Mapper (v1.0.0, Huerta-Cepas et al. 2017) was parsed by funannotate and combined with annotations made by using the following databases: Pfam (v32.0, El-Gebali et al. 2019), gene2product (v1.32, https://github.com/nextgenusfs/gene2product, last accessed February 8, 2021), dbCAN (v7.0, Huang et al. 2018), MEROPS (v12.0, Rawlings et al. 2018), UniProtKb (downloaded Feb 2019, The UniProt Consortium 2019). We predicted gene names and product descriptions were done by parsing UniProtKb and Eggnog-Mapper searches and cross-referencing results to gene2product database (v1.32). The details on how we used the funannotate pipeline for genome annotation can be found at github (https://github.com/metalichen/).

We analyzed the proteins predicted by funannotate using the KAAS webserver (Moriya et al. 2007). We used the antiSMASH web server (Blin et al. 2019) to detect secondary metabolite clusters. To build heatmaps of CAZy and MEROPS families across the three MAGs, we parsed the funannotate outcome using a custom R script. Subfamily-level CAZy annotations were collapsed. We used OrthoVenn webserver (Wang et al. 2015) to annotate orthologous clusters across the three fungal MAGs. To identify putative ribitol transporters, we followed (Armaleo et al. 2019). We ran BLASTp search against the predicted proteins using sequences of characterized sorbitol/mannitol/ribitol/arabitol/H+ symporters from *Debaryomyces hansenii* (NCBI Accession Numbers CAG86001 and CAR65543; Pereira et al. 2014) as a query.

To identify secreted proteins, we used a three-step process. First, all proteins were analyzed using SignalP (Bendtsen et al. 2004). All protein models estimated to have a secretion signal were then analyzed with the TMHMM web server (Krogh et al. 2001). Only models with secretion signal and no transmembrane domain were retained. However, we allowed one transmembrane domain in the N-terminal 60 amino acids, since it often corresponds to the secretion signal. Finally, this set of proteins were analyzed with WoLF PSORT (Horton et al. 2007); the final list only included models with >60% of nearest neighbors belonging to secreted proteins. We defined SSP as secreted proteins <300 amino acids (Pellegrin et al. 2015); putative effectors were identified using the EffectorP webserver (v2.0, Sperschneider et al. 2018).

For four protein families that we reported missing from individual fungal MAGs, we ran an additional search to check whether they are truly missing or were missed in our analysis due to imperfect binning or genome annotation. We used metaEuk (v2, Levy Karin et al. 2020) to predict proteins across all metagenomic contigs. We then ran hmmsearch (HMMER v3.2.1, Eddy 2011) with an *E*-value cutoff of 10*e*-5 to identify the following Pfams corresponding to the missing protein families: PF01083 for CAZY CE5, PF01670 for GH12, PF00089 for MEROPS S1, and PF01583 for adenylylsulphate kinase. We subsequently ran diamond blastp (Buchfink et al. 2015) against UniRef50 (UniProt 2020_02) with parameter -top 3 and used majority voting to identify eukaryotic hits. Among them, we selected hits associated with the studied MAGs: First identifying hits that landed on contigs assigned to these MAGs, then searching the remaining (unbinned) hits against UniRef50 and selecting those that returned fungal proteins. If our search yielded a candidate protein assignable to a MAG, we did not report this family missing.

For the comparative genomics study, we annotated fifteen additional genomes (supplementary table S8, Supplementary Material online). For each of them, we obtained nucleotide assemblies and annotated them in the same way as described above. We used the “funannotate compare” function to compare this set of genomes. “Funannotate compare” summarizes all functional annotations for the genomes; it also runs a phylogenomic analysis based on single-copy orthologs. Randomly selected BUSCO orthologs were concatenated for each genome, aligned using MAFFT and analyzed using RAxML using PROTGAMMAAUTO substitution model and 100 rapid bootstrap replicates.

### CAZyme Analysis

We calculated the distribution of different CAZy families in the three fungal MAGs using dbCAN annotations produced by funannotate. For this purpose, all annotations on the sub-family level were collapsed. Then, we isolated all CAZymes labeled as secreted proteins and analyzed them in the same way.

We selected families of interest and analyzed them in depth using SACCHARIS pipeline (Jones et al. 2018). Characterized GH5 full length sequences from these families were downloaded from the CAZy website and aligned with the CAZymes identified in the MAGs. Sequences were trimmed to the catalytic domains using dbCAN (Huang et al. 2018) and aligned with MUSCLE (Edgar 2004). The phylogenies were reconstructed using FastTree2 (Price et al. 2010) and visualized using iTOL (Letunic and Bork 2019).

## Results

### Extraction of Symbiont Genomes from Metagenomic Data

**Fig. 1.**
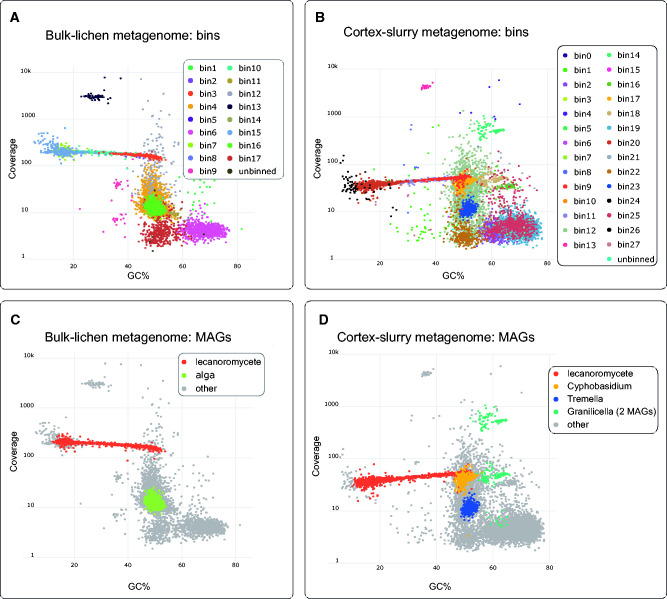
The assignment of contigs to bins and genomes in the two *Alectoria* lichen metagenomes. Bins and The *Alectoria sarmentosa* lichen and its metagenomes. (*A*) Bulk-lichen metagenome, colors assigned based on the initial binning. (*B*) Cortex-slurry metagenome, colors assigned based on the initial binning. (*C*) Bulk-lichen metagenome, colors represent MAG assignments. (*D*) cortex-slurry metagenome, colors represent MAG assignments. According to preliminary taxonomic assignment, bin 3 from the bulk-lichen metagenome, and bin 9 from the cortex-slurry metagenome were assigned to Ascomycota. Each of them was a part of a linear-shaped cloud extending from 10% to 55% of GC-content. In each metagenome separately, we merged bins constituting the linear cloud, which was additionally verified by the taxonomic placement of the bins. The bulk-lichen metagenome contained MAGs of the two core partners of the symbiosis, the lecanoromycete and the alga. The cortex-slurry metagenome, in addition to the lecanoromycete genome, contained MAGs of two SFSs and two bacterial MAGs.

We generated metagenomes from two samples of *A. sarmentosa*: One from pulverized bulk lichen material, and the other from pelleted sediment obtained by soaking a thallus in hot water (cortex slurry). We assembled each metagenome separately (supplementary table S1, Supplementary Material online). In order to separate symbiont genomes within metagenomes, we binned (or grouped) contigs using tetranucleotide frequency patterns and sequence coverage. For each bin, we assigned provisional taxonomic identifications by drawing 200 proteins at random and deriving taxon predictions from UniProt (see Materials and Methods). Next, we generated estimates of completeness and contamination for each bin as putative metagenome-assembled genomes (MAGs) and plotted the contigs as GC-coverage plots (fig. 1*A* and *B*). Ab initio binning and taxon assignment led to the recognition of two large eukaryotic genomes in the bulk lichen metagenome, corresponding to an ascomycete fungus and chlorophyte alga; and five in the cortex slurry, one from an ascomycete fungus, two from basidiomycete fungi, and two from bacteria (table 1). Of the two basidiomycete MAGs, one had completeness estimates varying, dependent on the tools employed, from 83.9% to 97.7%, the other from 83.4% to 90.7%. Estimated contamination rates were all below 1% (table 1). The algal MAG was nearly complete but had a contamination rate of 80% (supplementary table S2, Supplementary Material online); no algal MAG was recovered from the cortex slurry. Each of these MAGs was recovered as a single bin.

The highest coverage MAG in both metagenomes belonged to an ascomycete (supplementary table S2, Supplementary Material online). However, the bin identified as the core ascomycete MAG in both of the metagenomes had completeness of only 80–92%, as reported by different tools, despite high coverage (supplementary table S3, Supplementary Material online). That being said, we noticed several bins arranged at near-identical coverage to the ascomycete bin in the GC-coverage plots, forming a more or less linear cloud, ranging in GC content from ∼30% to 55% (fig. 1*A* and *B*). To explore the possibility that these additional bins also belonged to, and would complete, the ascomycete MAG, we inferred their taxonomy. For each of the eight bins, the inferred lineage was Ascomycota (supplementary table S2, Supplementary Material online). Merging these four bins (fig. 1*C* and *D*) improved completeness for the ascomycete MAG to around 98% whereas not significantly impacting estimated contamination (by <1%; supplementary table S3, Supplementary Material online). For the downstream analysis, we treated the merged bins as a single MAG.

### Symbiont Genomes from *Alectoria* Metagenomes

**Fig. 2.**
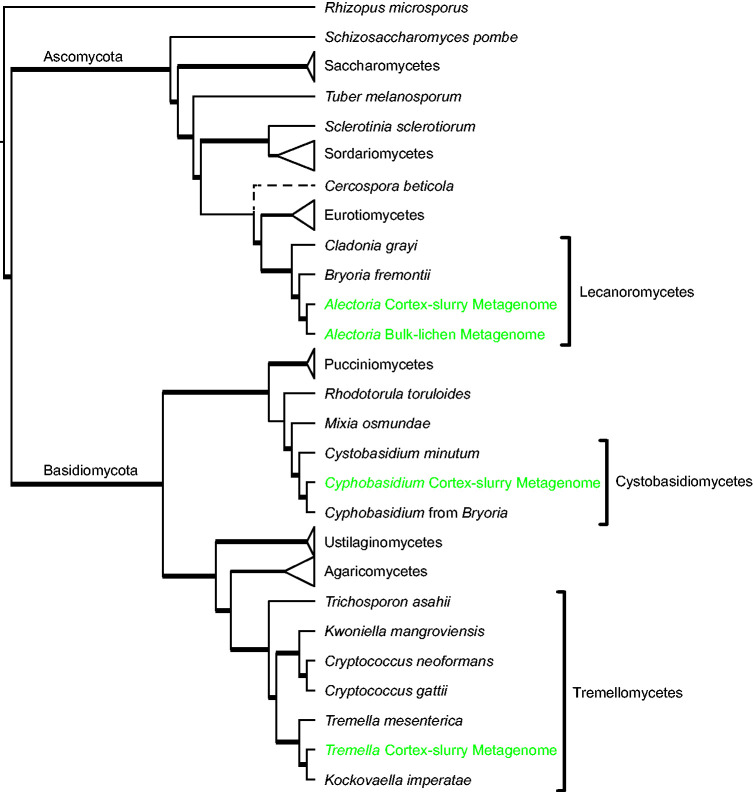
Maximum likelihood phylogenomic tree based on 42 fungal proteomes and 71 single-copy orthologous loci. Data derived from the studied metagenomes are indicated in green. Bold lines indicate ASTRAL bootstrapping >90 (species tree) based on 1000 bootstrap replicates per gene, and IQTREE ultrafast bootstrap >95 (concatenated tree) based on 1000 replicates. The dashed line indicates a conflict between the species tree and concatenated tree.

The cortex-slurry metagenome yielded five nearly complete MAGs, three fungal and two bacterial (table 1). In order to refine the taxonomic placement of the fungal MAGs, we performed a phylogenomic analysis based on 71 single copy orthologs identified in 38 published fungal genomes and all the fungal MAGs from both metagenomes. Using this approach, we placed the ascomycete MAG from both metagenomes in the class Lecanoromycetes, confirming its identity as the dominant fungus of the lichen symbiosis (fig. 2). For clarity, this fungus, which formally carries the name *A. sarmentosa* under the code of nomenclature, will be hereafter called the “lecanoromycete” whereas the lichen itself will be referred to as the “*Alectoria* lichen.” The remaining two MAGs resolved within the basidiomycete classes Cystobasidiomycetes and Tremellomycetes, respectively (fig. 2). The cystobasidiomycete is an exact match for a known, unnamed *Cyphobasidium* species previously detected by PCR from *Alectoria* lichen (hereafter *Cyphobasidium*; supplementary fig. S1, Supplementary Material online). The tremellomycete is newly detected in the *Alectoria* lichen and is sister to *Biatoropsis usnearum*, a member of *Tremella* s.lat. (hereafter *Tremella*; Millanes et al. 2011) (supplementary fig. S2, Supplementary Material online). Using CheckM (Parks et al. 2015), both bacterial MAGs isolated from the cortex-slurry metagenome were assigned to *Granulicella* (Acidobacteria; supplementary table S4, Supplementary Material online).

The use of cortex slurries led to a significant change in symbiont DNA, and considerably increased the coverage of secondary symbionts. In the bulk-lichen metagenome, both *Cyphobasidium* and *Tremella* were also present, as was shown by the presence of their rDNA sequences. But their coverage was insufficient for them to be assembled and recovered as identifiable bins. Coverage of a contig containing the ITS of *Cyphobasidium* was 336 times lower than that of the dominant fungus; for *Tremella* the ratio was 1:184. In the cortex-slurry metagenome, the same ratios were 1:4 for *Cyphobasidium* and 1:5 for *Tremella*, constituting an 84-fold and 36-fold coverage increase, respectively.

The basidiomycete MAGs were less than half as large as the lecanoromycete MAG (table 1); GC content was 38% for the lecanoromycete and 51–52% for the basidiomycetes. De novo genome annotation resulted in 9,407 protein-coding gene models for the main fungus, 6,095 for *Cyphobasidium*, and 6,038 for *Tremella* (supplementary table S5, Supplementary Material online). A gene prediction based on known orthologs could be modeled for only a portion of them (64–71%; see Materials and Methods). A large suite of functional elements was shared between ascomycete and basidiomycete (supplementary fig. S3, Supplementary Material online).

### Constancy of Association

**Fig. 3.**
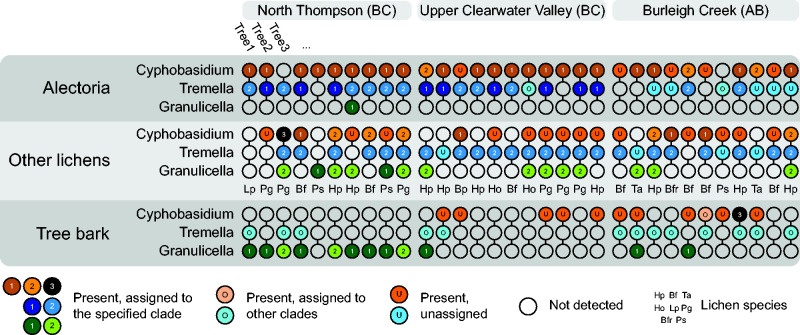
Frequency of association of the three low-abundance partners identified in the cortex-derived metagenome based on PCR-screening of *Alectoria* lichen thalli paired with a random non-*Alectoria* lichen and tree bark from the same branch on 32 trees from three localities in British Columbia and Alberta, Canada. Each vertical column represents one sample tree. Colored circles represent presence; numbers 1–3 correspond to the clade the sequence was recovered from (see supplementary figs. S4–S6, Supplementary Material online for the phylogenetic trees), “O” are sequences recovered in other parts of the tree, “A” are sequences unassigned due to poor quality (identity of these was sequences verified by searching them against NCBI). Letter codes stand for species of associated macrolichens used for assays: Bfr, *Bryoria fremontii*; Bf, *Bryoria fuscescens*; Ho, *Hypogymnia occidentalis*; Hp, *Hypogymnia physodes*, Lp, *Lobaria pulmonaria*, Ps, *Parmelia sulcata*; Pg, *Platismatia glauca*; Ta, *Tuckermannopsis americana*.

Only one of the two basidiomycete fungi and none of the bacteria had previously been reported as *Alectoria* lichen symbionts. In order to assess whether these occur as stably associated symbionts, we used PCR to screen for their presence in 32 thalli of *Alectoria* lichens from three locations in eastern British Columbia and western Alberta. In each case, the sampled *Alectoria* thallus was sampled together with a randomly chosen, adjacent lichen symbiosis and adjacent bare bark on the same tree. All *Alectoria* thalli contained at least one SFS; most contained both *Cyphobasidium* and *Tremella* (fig. 3, supplementary tables S6 and S7, Supplementary Material online).

Most sequences of *Cyphobasidium* and *Tremella* from *Alectoria* lichens, including sequences extracted from the metagenomes, were recovered in known lichen-associated clades of these two genera (supplementary figs. S4 and S5, Supplementary Material online). Most *Cyphobasidium* from *Alectoria* formed a clade mixed only with *Cyphobasidium* from closely related *Bryoria* lichens (clade 1, fig. 3, supplementary fig. S4, Supplementary Material online); a few sequences came from clade 2, made up by Cyphobasidiales from other lichen symbioses. In *Tremella* from *Alectoria*, by contrast, a much larger percentage of samples drew from a clade shared with other lichen symbioses: Half of the sequences formed their own clade (clade 1, fig. 3, supplementary fig. S5, Supplementary Material online), whereas half came from the clade 2, which also constituted the majority of the sequences we obtained from other lichens. The sequenced MAGs of *Cyphobasidium* and *Tremella* belong to clade 1 on their respective trees (supplementary figs. S4 and S5, Supplementary Material online).

Both SFS lineages also occurred in some other lichens, and occasionally in bark samples. By contrast, we found *Granulicella* in only one *Alectoria* thallus (fig. 3), but 12 bark extractions. We concluded that this bacterium is not stably associated with the *Alectoria* lichen and excluded it from further analyses.

### The Basidiomycete MAGs Are Similar to Closely Related Genomes but Have Smaller Secretomes

**Fig. 4.**
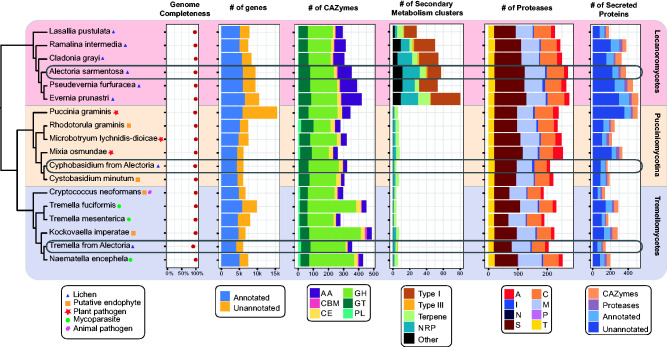
Comparative genomic analysis of the three fungi from *Alectoria* lichen with closely related genomes. Maximum likelihood phylogeny based on 500 loci was juxtaposed with the genome-level comparisons of number of genes, carbohydrate-active enzymes (CAZymes), secondary metabolism gene clusters (SMGC), proteases, and secreted proteins across the twelve genomes. Classes of CAZymes included auxillary activity enzymes (AA), carbohydrate-binding modules (CBM), carbohydrate esterases (CE), glycoside hydrolases (GH), glycosyl transferases (GT), and polysaccharide lyases (PL). SMGCs included various polyketide synthases (PKS), nonribosomal peptide-synthetases (NRPS), terpene synthases and other. Protease classes included aspartic peptidases (A), cysteine peptidases (C), metallopeptidases (M), asparagine peptidases (N), mixed peptidases (P), serine peptidases (S), threonine peptidases (T), and protease inhibitors (I). Genome completeness was calculated using EukCC. We counted proteins as unannotated if they had no UniProt, Pfam, dbcan, or MEROPS annotation.

As a “sanity check,” we compared all three of our MAGs with genomes sequenced from cultures of closely related species. All three MAGs were similar to related genomes in gene count, assembly size and GC content (fig. 4, supplementary table S8, Supplementary Material online). The MAG of the *Alectoria* lichen lecanoromycete compared with five other lecanoromycete genomes, all of which are lichen fungal symbionts, exhibited numbers of Carbohydrate Active enZymes (CAZymes), secondary metabolite gene clusters (SMGC) and secreted proteins close to average among the six genomes (354 CAZymes, 57 SMGC, and 374 secreted proteins in the *Alectoria* lichen lecanoromycete vs. 346 CAZymes, 54 SMGC, and 372 secreted proteins on average; fig. 4).

The basidiomycete fungi from the *Alectoria* lichen were similar to their close relatives in the SMGC and CAZyme profiles (fig. 4). All twelve studied genomes, with one exception, harbored several putative SMGCs belonging to nonribosomal peptide synthetases (NRPS) and terpene classes. *Tremella* from the *Alectoria* lichen was the only genome to include a polyketide synthase (PKS) cluster. Numbers of CAZymes in both basidiomycetes were close to average (322 in *Cyphobasidium* and 356 in *Tremella* vs. 344 on average). We compared CAZyme profiles of fungi with different ecology (e.g., plant pathogens and mycoparasites) but failed to detect any lifestyle-dependent pattern (supplementary table S9, Supplementary Material online). The most notable difference is in the size of secretomes, which were smaller in both of the lichen-associated basidiomycetes compared with their relatives. This observation is unlikely to be fully explained by potential incompleteness of the MAGs, as not only the number of genes identified as secreted, but also their percentage across all genes were lower in the MAGs than in the related genomes (2.8% in *Cyphobasidium* vs. 3.4% on average among Pucciniomycotina; 2.4% in *Tremella* vs. 2.7% on average among Tremellales).

### The Three Fungal Genomes Show Evidence of Different Cell Wall and Secreted Polysaccharide Profiles

Our genomic evidence was consistent with data on cell walls of fungi related to the three studied species. Putative chitin and β-1,3-glucan synthases (*GAS1*, *CHS1*, *CHS2*, *CHS3*, *CHS5*, *CHS7*; Lesage et al. 2004, 2005) found in the lecanoromycete matched the reports of chitin and glucan (reviewed by Spribille et al. 2020). The cell walls of *Cryptococcus neoformans*, a close relative of *Tremella*, are built by α-1,3 and β-1,3-glucans, chitin, and chitosan (Doering 2009). In the *Tremella* MAG we identified genes involved in biosynthesis of all of these polysaccharides: Putative α-1,3-glucan synthase *AGS1*, β-1,3-glucan synthase *FKS1* (Lesage et al. 2004), chitin synthases (*CHS1*, *CHS2*, *CHS3*, *CHS5*, *CHS7*; Lesage et al. 2005), as well as putative chitin deacetylase CDA2, which catalyzes deacetylation of chitin into chitosan (Martinou et al. 2003). For the class Cystobasidiomycetes, only the monosaccharide composition of the cell wall is known (Takashima et al. 2000). The presence of putative β-1,3-glucan synthase *FKS1* and putative chitin synthases (*CHS1*, *CHS2*, *CHS3*, *CHS7*) in the *Cyphobasidium* MAG suggested that the cell wall composition includes β-1,3-glucans and chitin.

The extracellular polysaccharides reported from lichens similar to *Alectoria* include variously-linked glucans (β-1,3; β-(1,3),(1,4); α-(1,3),(1,4)) and heteromannans, predominantly with α-1,6-mannan backbones (Spribille et al. 2020). We identified genes potentially involved in the synthesis of these polysaccharides in all three fungi. Putative β-1,3-glucan synthases were found in all three MAGs. Based on this, all three fungi seemed equally likely to produce β-(1,3),(1,4)-glucans. By contrast, only the lecanoromycete and *Tremella* possessed putative α-1,3-glucan synthase *AGS1*, even though all three fungi had an enzyme making putative α-1,4 bonds (*GSY1*). The lecanoromycete was also unique in containing proteins similar to those from the mannan polymerase complex II, which synthesizes α-1,6-mannan backbone in multiple ascomycete fungi (e.g., Henry et al. 2016). Although all three MAGs encoded some GT32 enzymes known to be involved in α-1,6-mannan biosynthesis, the lecanoromycete had more than either of the basidiomycetes (supplementary fig. S7, Supplementary Material online).

**Fig. 5.**
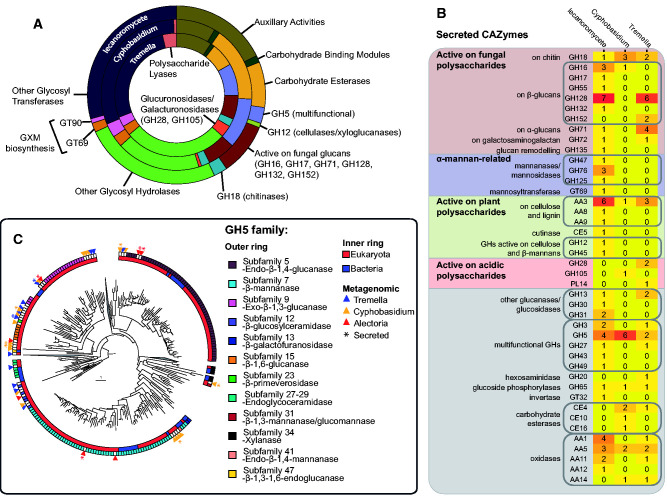
Carbohydrate-active enzymes (CAZymes) in the three fungal MAGs. (*A*) Relative abundance of major groups of CAZymes in the three MAGs, highlighting specific families of glycoside hydrolases and glycosyl transferases discussed in the text. (*B*) Heatmap of CAZyme families predicted to be secreted by the three fungi, grouped by major types of activity. (*C*) Results of the SACCHARIS analysis of GH5 enzymes from the studied MAGs, showing the position of GH5 enzymes identified in the studied MAGs (indicated with triangles) in relation to characterized GH5s. Secreted proteins are indicated with an asterisk. Color rings are assigned based on the primary subfamily enzymatic activity and origin (bacterial vs. eukaryotic).

The genomic data predicted the synthesis of several acidic polysaccharides not yet reported from lichens. First, glucuronoxylomannan (GXM) is a polysaccharide known from *Cryptococcus* (e.g., Zaragoza et al. 2009) and, in the form of a GXM-like polysaccharide that includes fucose, in non-lichen *Tremella* (de Baets and Vandamme 2001). Both *Tremella* and *Cyphobasidium*, but not the lecanoromycete, contained homologues of all so-called CAP genes (*CAP10*, *CAP59*, *CAP60*, and *CAP64*), which play a role in capsule synthesis in *Cryptococcus* (Zaragoza et al. 2009). Only *CAP10* (CAZy family GT90) was present in the lecanoromycete. It also possessed four proteins assigned to the same GT69 family as *CAP59* and *CAP60* (fig. 5*A*). Consistent with a fucose-containing polysaccharide, both basidiomycete MAGs but not the ascomycete code for putative GDP-L-fucose synthase *GER1* (1.1.1.271). Second, we also found two GT families involved in heparan sulfate biosynthesis, GT47 and GT64, in the basidiomycete MAGs (supplementary fig. S7, Supplementary Material online). Currently, the only fungal GT47 enzyme is reported from *C. neoformans* (Geshi et al. 2018). GT64s have been reported from other fungi only a few times (Chang et al. 2016). As heparan sulfate production is not known from any fungus, it may play a role in producing an acidic polysaccharide that displays different monosaccharide composition of linkages, as suggested by Grijpstra (2008) for cryptococcal GT47. Third, the inferred ability of the lecanoromycete to produce glucuronic acid stood in contrast to previous reports where uronic acids were reported missing from cultures of some lecanoromycetes (Honegger and Bartnicki-Garcia 1991).

### The Lecanoromycete Genome Codes for More Degradative Enzymes That Target Plant Polysaccharides than Either SFS (fig. 5*B*)

Among GH5 predicted to be secreted by the lecanoromycetes and *Cyphobasidium*, we identified enzymes from subfamilies GH5_7 (β-mannanases) and GH5_5 (β-1,4-glucanases). Figure 5*C* shows a GH5 family tree that was used to infer functions of the GH5 from the studied MAGs. These enzymes were identified as targeting plant polysaccharides, because the corresponding substrates (β-mannans and β-1,4-glucans, such as cellulose) are components of the plant cell wall (Burton et al. 2010) and not known to be produced by the studied fungi. It is possible that these enzymes are used to hydrolyze components of the algal cell wall, which was shown to contain polysaccharides with these structures (Centeno et al. 2016). The lecanoromycete MAG was the only one to code for a putative secreted glucanase or xyloglucanase from the GH12 family (fig. 5*A* and *B*), which might target cellulose and is known to be upregulated in lecanoromycete-alga coculturing experiments (Kono et al. 2020), and a secreted β-mannanase or β-1,4-glucanase from the GH45 family. Some secreted auxiliary activity CAZymes (AA) belonged to families likewise involved in digesting plant polymers through oxidative processes: AA3 (active on cellobiose and lignin) in all three secretomes and AA9 (active on cellulose) in the lecanoromycete secretome. The lecanoromycete MAG also coded for a putative secreted cutinase (carbohydrate esterase CE5, Pfam accession PF01083), which targets plant cuticle (Nakamura et al. 2017; supplementary fig. S7 and table S10, Supplementary Material online).

### The Lecanoromycete Genome Codes for More Secondary Metabolite Clusters than Either SFS

*Alectoria* lichen produces usnic acid, α-alectoronic acid and barbatic acid (Brodo and Hawksworth 1977). Both α-alectoronic and barbatic acid are biosynthetically related compounds derived from the polyketide orsellinic acid. Orsellinic acid has been linked to a Group I nonreducing Type I PKS (Liu, Zhang, et al. 2015), an apparent ortholog of which was present in the lecanoromycete (62% identity over 99% query cover). Usnic acid is a dibenzofuran derived from orsellinic acid, though evidence has recently been advanced to suggest a nonreducing PKS gene cluster including methylphloracetophenone synthase and methylphloracetophenone oxidase correlates with the upregulation of usnic acid (Abdel-Hameed et al. 2016). An orthologue of this PKS cluster, too, was found in the lecanoromycete (84% identical over 99% cover). In the *Alectoria* lichen, the majority of SM clusters and all but one PKS cluster were found in the lecanoromycete (fig. 4, supplementary table S5, Supplementary Material online). We found far more SMGCs than there are known secondary metabolites in the *Alectoria* lichen (57 SMGCs vs. three secondary metabolites).

Among SMGCs predicted for the lecanoromycete, two showed similarity to characterized clusters producing toxins. In a NRPS cluster, the core biosynthetic gene was similar to one from the aspirochlorine gene cluster, a mycotoxin known from *Aspergillus* (57% identity of the amino acid sequence, over 95% query coverage). A terpene gene cluster showed similarity to the gene cluster producing PR-toxin (62% identity, over 97% query coverage), a mycotoxin from *Penicillium*. A gene similar to fusarin synthase was assigned to the same cluster (46% identical over 93% query cover).

We found fewer predicted SMGCs in *Cyphobasidium* and *Tremella* (fig. 4). All but one SMGC found in the basidiomycetes were NRPS and terpene clusters. A Type III PKS cluster predicted in *Tremella* was the only PKS cluster in the basidiomycetes.

### SFSs Genomes Predict Nutrient Limitation and Scavenging

Putative secreted phosphorus-scavenging enzymes are more numerous in the basidiomycete MAGs than in the lecanoromycete (supplementary table S10, Supplementary Material online). Both basidiomycete secretomes contain purple acid phosphatase-like proteins, a type of acid phosphatase known mostly from plants and some ascomycete fungi: Two proteins in *Cyphobasidium* (Pfam accession PF16656 and PF14008) and one in *Tremella* (PF14008). Histidine phosphatase superfamily branch 2 contains some enzymes that break down nucleotides and phytic acid. These enzymes are secreted by fungi for scavenging phosphorus from extracellular sources (Rigden 2008). We found two similar proteins (PF00328) in *Tremella* and one in the lecanoromycete. The three fungi had a similar set of putative phosphate transporters (*PHO84* and *PHO91*), but in *Tremella* *PHO84* appeared duplicated.

The *Tremella* MAG lacked some nutrient assimilation enzymes, suggesting it is auxotrophic. Through KEGG annotation, we found key enzymes (nitrate transporter, nitrate reductase, and nitrite reductase) in the nitrogen assimilation pathway in the lecanoromycete and *Cyphobasidium*, but *Tremella* lacked all three. This is consistent with reports that some members of Tremellales are unable to assimilate nitrate or nitrite as nitrogen sources (Lee et al. 2011).

### The Lecanoromycete Exhibits More Pathogenic Features than Either Basidiomycete

Numerous studies have undertaken to connect fungal lifestyle to genomic signatures (e.g., Pellegrin et al. 2015). The leading candidates that have been studied are proteases, polysaccharide lyases, glycoside hydrolases (GH) and lipases. Each of these is represented in all three of the *Alectoria* lichen fungal genomes, in differing proportions. The lecanoromycete secretome contained twice as many proteases as that of *Tremella* and almost three times as many as in *Cyphobasidium* (fig. 4, supplementary table S5, Supplementary Material online). This increase is proportionate to the secretome size. Only the lecanoromycete contained trypsin-like proteases (MEROPS family S1) (supplementary fig. S8, Supplementary Material online), associated with pathogenic fungi regardless of their host (Dubovenko et al. 2010). Subtilisin proteases (S8), known to be involved in mycoparasitism (Fan et al. 2014) were present in a greater number in the lecanoromycete MAG, but only *Tremella* subtilisins were predicted to be secreted.

In endophytes and plant pathogens, fungalysin, a metalloprotease (M36), plays a role in suppressing host defenses by cleaving chitinases released by the plant in response to fungal infection (Zuccaro et al. 2011, Sanz-Martín et al. 2016). Both *Tremella* and the lecanoromycete MAGs encoded fungalysin, but only the lecanoromycete fungalysin was predicted to be secreted (supplementary fig. S8, Supplementary Material online). The lecanoromycete also was the only fungus to have two other secreted proteins that in fungi suppress chitin-triggered immune response: LysM domain-containing protein (PF01476), which binds to chitin to mask it from host immune systems (Kombrink and Thomma 2013); and a chitin-binding protein (PF00187; supplementary table S10, Supplementary Material online).

The numbers of putative secreted lipases predicted in the three fungi are low. The lecanoromycete secretome contained three lipases assigned to four Pfam families (accessions PF01764, PF01735, PF03893, and PF13472, respectively) whereas the basidiomycete MAGs encoded one secreted lipase-like protein each (supplementary table S10, Supplementary Material online). A phospholipase-like domain PLA2_B (PF04800) found in *Tremella* was also present in the lecanoromycete. A GDSL-like lipase/acylhydrolase (PF00657) was found only in *Cyphobasidium*. Secreted lipases, whereas known from mutualistic fungi (Chen et al. 2018), are thought to contribute to pathogen virulence (Pellegrin et al. 2015).

The only secreted protease inhibitor, from MEROPS family I51, was encoded in the *Cyphobasidium* MAG. Members of this family act as inhibitors of serine carboxypeptidases Y (S10), of which the lecanoromycete possessed the largest number that were predicted as secreted, though they were predicted from all three fungi.

### We Found No Evidence of Any of the Fungi Targeting Polysaccharides Produced Exclusively by Other Fungal Partners

For all three fungi, the majority of secreted GH appeared to be active on polysaccharides synthesized by the same fungus, including β-glucanases (GH128, GH16, GH17, GH132, GH152, some GH5) and chitinases (GH18; fig. 5*B*). The same two MAGs that encoded putative α-1,3-glucan synthase, *Tremella* and the lecanoromycete, were predicted to secrete α-1,3-glucanase (GH71). Similarly, all CAZy families targeting acidic polysaccharides (GH28, GH105, polysaccharide lyase PL14) were predicted to be secreted by the basidiomycetes, which are predicted to synthesize acidic polysaccharides. We did not identify any GHs that definitively target polysaccharides produced by other fungal members of the symbiosis in any pairwise combination.

### All Three Fungi Possess Predicted Polyol Transporters

In each of the three fungi, we found a protein highly similar to characterized d-sorbitol/d-mannitol/ribitol transporters (BLASTp *e*-value < 1*e*-140). All three proteins were assigned to PF00083 (Sugar [and other] transporter). All three possessed several transmembrane domains, though only the protein from *Cyphobasidium* possessed twelve transmembrane domains, as is typical for sugar transporters (Leandro et al. 2009), whereas proteins from the lecanoromycete and *Tremella* had seven and eight, respectively.

### We Cannot Rule Out or Confirm That Any of the Fungi Are Oleaginous

As both basidiomycetes have relatives within the same class that produce large amounts of lipids (oleaginous fungi; Sitepu et al. 2014), we examined the MAGs for the presence of genes known to be involved in lipid production following Beopoulos et al. (2009) and Adrio (2017). In fact, from all three fungi we predicted most of the enzymes required for being oleaginous: 1) enzymes involved in lipid biosynthesis initiation: AMP deaminase *AMD1*, ATP-citrate lyase *ACL1*, malic enzyme *MAE1* (also called *MDH1*), and acetyl-CoA carboxylase *ACC*; 2) fatty acid synthases *FAS1* and *FAS2*; and 3) enzymes involved in triacylglycerol synthesis: glycerol-3-phosphate acyl transferase (*SCT1*, EC 2.3.1.15 identified by KEGG Pathway annotation), lysophosphatidic acid acyltransferase (*SLC1*, EC 2.3.1.51), phosphatidic acid phosphohydrolase (*PAP*, EC 3.1.3.4), and diacylglycerol acyltransferases *DGA1* and *LRO1* (EC 2.3.1.158). However, the key enzyme for steryl ester synthesis, sterol O-acyltransferase (*ARE1* and *ARE2*, EC 2.3.1.26), was predicted only for the lecanoromycete and *Cyphobasidium*.

### All Three Fungi Have Machinery for Dimorphic Switching

In the three fungi, we searched for the homologs of genes regulating dimorphic switching in other fungi, originally characterized from *Candida albicans*, the yeast-to-hypha switching of which is well characterized (Sudbery 2011). Dimorphic switching in fungi is controlled through cAMP/PKA and MAPK pathways (Borges-Walmsley and Walmsley 2000). In all three fungi, we found the key enzymes involved in this process: Adenylate cyclase *CYR1*, small G proteins *RAS2*, *GPA2*, and *CDC42*, protein kinase A *PKA*, p21-activated kinase *STE20*, and elements of MAPK cascade *STE11*, *STE7*, and *STE2*. Downstream targets of the signaling pathways are transcriptional factor pathways (Borges-Walmsley and Walmsley 2000). The only protein identified as associated with the yeast-form growth in the lecanoromycete from Park et al. (2013) was a C2H2-type zinc finger transcription factor (Jeong et al. 2015), a type of transcription factor common across eukaryotes (Wolfe et al. 2000). We found multiple C2H2 zinc finger domain-containing proteins (PF00096) in all three fungal MAGs. Similar proteins had been already reported as dimorphic transition regulators in other fungi (Hurtado and Rachubinski 1999), and a C2H2-type zinc finger transcription factor was reported before as a suppressor of hyphal growth in *C. albicans* (Murad et al. 2001). Of transcription factors suppressing hyphal growth, two (*RFG1*, identified through KEGG annotation, and *TUP1*) were predicted in the lecanoromycete and *Tremella* (Kadosh and Johnson 2001). Among other genes playing the same role, *NGR1* was predicted in *Tremella*, and *SSN6* and *TEC1* (identified through KEGG annotation) were predicted in *Cyphobasidium*. Transcription factors promoting hyphal growth were predicted from all three MAGs with the lecanoromycete having the most: *SKN7* and *CRZ1* in all three fungi, *STE12* in the lecanoromycete and *Tremella*, *ACE2* in the lecanoromycete and *Cyphobasidium*, *EFG1*, *CSR1* and *UME6* in the lecanoromycete, and *FLO8* in *Cyphobasidium*.

## Discussion

Our study is the first to provide genome annotations of SFSs in a lichen and the first to compare and contrast the potential of primary and secondary fungal symbionts. The genomes of SFSs we describe here possess far fewer genes than the lecanoromycete, and rank within the smallest 5% of 1,737 sequenced fungal genomes to date (https://mycocosm.jgi.doe.gov/fungi/fungi.info.html, last accessed February 8, 2021). Though genomic data will ultimately need to be complemented with other lines of evidence, patterns of gene enrichment and secretion provide clear evidence of divergent function and inform previous hypotheses of lifestyle among the three fungi in the *Alectoria* lichen. These results are furthermore robust to the possibility of false absence of one or few genes. Two of the three MAGs, the lecanoromycete and *Cyphobasidium*, are >97% complete; the *Tremella* MAG is only ∼90% complete, but still within the threshold commonly used in metagenomics (Bowers et al. 2017) and high compared with other published eukaryotic MAGs (Delmont et al. 2018). It is therefore unlikely that, for example, CAZyme profiles of the fungi will significantly change.

### Potential Contributions of the Fungal Partners

Even with these limitations, however, three clear patterns stand out from our comparison of the three genomes. First, our data are consistent with the theory that SFSs produce secreted polysaccharides that can contribute to the extracellular matrix. Most lecanoromycete-derived lichens possess α-1,6-mannans (Spribille et al. 2020), a common product of ascomycetes (Leal et al. 2010), and our genomic data confirmed that these can be produced by the lecanoromycete. It is however not clear if or to what extent α-1,6-mannans account for the extracellular matrix that holds fungal cells together in the form of a lichen. Acidic polysaccharides are known to be a part of this matrix based on histological studies (e.g., Modenesi and Vanzo 1986), but acidic polysaccharides have never been experimentally assessed in lichens and are basically a black box (Spribille et al. 2020). Of the SFSs, *Tremella* is closely related to species that produce copious, capsular, GXM-like polysaccharides characterized by possessing α-1,3-mannan backbones. Several genes have been identified as related to α-1,3-mannan capsule production in *C. neoformans*, and we found putative orthologs of all of these, not only in the *Tremella* MAG but also in the *Cyphobasidium* MAG. Representatives of the same CAZyme families, though not direct *Cryptococcus* orthologs, are also found in the lecanoromycete. Interestingly, all three MAGs appear to code for genes that synthesize glucuronic acid, even though no lecanoromycete-derived polysaccharide with glucuronic acid has been experimentally isolated. In summary, this suggests that both *Cyphobasidium* and *Tremella* produce GXM-like molecules, but that some yet-to-be-detected polysaccharides from the lecanoromycete may also carry acidic residues.

Second, both SFS MAGs code for more phosphorus scavenging enzymes than the lecanoromycete, suggesting that these fungi might play a role in lichen nutrient acquisition. Basidiomycete mutualists in general often provide this function to their plant partners, both in arbuscular and ectomycorrhizal relationships (Smith et al. 2011; Becquer et al. 2014). Phosphorus provision, and potential phosphorus limitation, is poorly understood in lichen systems, but notably *A. sarmentosa* has been shown to be P-limited under experimental conditions (Johansson et al. 2011).

Third and finally, our data clearly show that the lecanoromycete is the secondary metabolite cluster powerhouse of the *Alectoria* lichen. The close positive correlation of *Cyphobasidium* yeast abundance with an extracellular secondary metabolite, vulpinic acid (Spribille et al. 2016), appeared to suggest SM production either directly as a product of the SFSs or as the result of an interaction between fungi. Although we cannot address this specific SM with the data from the *Alectoria* lichen (which does not produce vulpinic acid), our data do appear to rule out the possibility that *Cyphobasidium* is producing PKS-derived SMs, such as those that dominate the *Alectoria* lichen (*Tremella* possesses one PKS cluster compared with 18 in the lecanoromycete). However, it is not clear that any of the SM clusters in the lecanoromycete can be connected with certainty to the synthesis of a known product. Crucially, our data cannot resolve the question, first advanced by Ahmadjian (1993) in a fungal–algal context, whether lichen SM precursors may be modified to form specific end products by mosaic pathways. There are precedents for SM end products derived from an orsellinic acid precursor, as several of the *Alectoria* lichen SMs are, to be produced only in coculture of fungi and bacteria (Schroeckh et al. 2009).

*Cyphobasidium* was first detected in the *Alectoria* symbiosis based on samples from Alaska, British Columbia, and Sweden (see table S7 in Spribille et al. 2016). In the present study, we confirmed the presence in high frequency of both *Cyphobasidium* and *Tremella* in *Alectoria* thalli in different geographic localities. This is the second lichen symbiosis, after *Letharia vulpina*, in which we have found representatives of both of these genera co-occurring over a wide geographic area (Tuovinen et al. 2019). Like in *L. vulpina*, we occasionally detected only one of the two symbionts in *Alectoria*. The similarity in their secretomes raises the intriguing possibility that they may be functionally redundant, which would be consistent with our finding of one SFS but not the other in about one fifth of the thalli sampled (fig. 3).

### The Dimorphism Wildcard

Of the three fungi in the *Alectoria* lichen, the two SFSs come from species groups known to routinely occur in both a hyphal and yeast stage, both of which can manifest themselves in the lichen thallus (Spribille et al. 2016; Tuovinen et al. 2019). The lecanoromycete is known to occur in the lichen symbiosis and by virtue of its sexual reproduction by ascospores is horizontally transmitted, and therefore must have an aposymbiotic life stage. At this point, however, nothing is known about this stage, and the fungus that occurs in the lichen is filamentous. Recently, Wang et al. (2020) confirmed dimorphism and the formation of a yeast stage, as well as the role of the PKA-cAMP pathway in regulated dimorphic switching, in the lecanoromycete *Umbilicaria muhlenbergii*. Our data show that the *Alectoria* lichen lecanoromycete likewise possesses cellular machinery for dimorphic switching. While this does not allow us to establish whether dimorphic switching actually occurs, it highlights how little is known about the life stage between sexual sporulation and reestablishment of the symbiosis to form a new lichen.

The gap in our knowledge about the aposymbiotic life stage for lecanoromycete lichen symbionts suggests we should use caution when trying to interpret the functions some of the genes the lecanoromycete MAG codes for. The lecanoromycete MAG codes for a suite of CAZymes targeting plant polymers. Some of these may occur in the algal cell walls (e.g., cellulose and β-mannans; Honegger and Brunner 1981; Centeno et al. 2016). Cutin, by contrast, is not known from green algae (Philippe et al. 2020). A qPCR-based study showed a predicted lecanoromycete cutinase orthologue to be expressed at similar levels in both axenic culture and during coculturing of the two dominant lichen symbionts (Joneson et al. 2011). The lecanoromycete also possesses numerous features more usually associated with pathogenic fungi. It has more secreted proteases, lipases and catabolic CAZymes than either of the SFSs, and is the only one that is predicted to produce toxins. Whether these enzymes are used to process secretions of the algal symbiont or are deployed in other settings remains to be tested. Finally, the lecanoromycete codes for far more SM clusters than it has documented SMs, a situation similar to *Cladonia uncialis* (Bertrand et al. 2018). This suggests either that many SMs are synthesized in quantities below detection thresholds, or alternatively in settings other than those that have been sampled.

### Can Genomic Data Reveal Signatures of Mycoparasitism?

When describing *Cyphobasidium* as a new genus, Millanes et al. (2016) speculated that the fungus is in fact a mycoparasite on the filamentous lecanoromycete in lichens. This they inferred from the occurrence of *Cyphobasidium* in the phylogenetic vicinity of other presumed mycoparasites in the Pucciniomycotina. The presence of genes coding for β-mannanases in the *Cyphobasidium* MAG strongly suggests that it may directly interact with plant cell walls, perhaps those of the symbiotic alga, at some point in its life cycle. Extrapolations regarding trophic relationships such as mycoparasitism—and their perpetuation in the literature—are common (e.g., Oberwinkler 2017), but experimental evidence is scarce. *Tremella lethariae*, originally presumed to be a mycoparasite of the lecanoromycete *L. vulpina* (Millanes et al. 2014), has been shown to enmesh algal cells (Tuovinen et al. 2019). Direct evidence of mycoparasitism, by contrast, has yet to be found in any lichen-associated *Cyphobasidium* or *Tremella* species, but studies to date have been limited.

The use of genomic data to infer mycoparasitism is hindered by the fact that fungal–fungal interactions are far less studied than fungal–plant interactions. Like plant pathogens, mycoparasites use secreted lytic enzymes during host invasion, but studies to date have not been able to find a consistent genomic signature for this. For example, a comparative genomic study did not show any enrichment in lytic enzymes in two mycoparasitic species within the ascomycete class Dothideomycetes (Haridas et al. 2020). Although the genomes of three mycoparasitic Tremellales, *Naematella encephela*, *Tremella fuciformis*, and *Tremella mesenterica*, have been sequenced, the molecular mechanisms of *Tremella*–host interactions remain undescribed. Kues and Ruhl (2011) hypothesized that ascorbate oxidase present in genomes of several mycoparasitic fungi, including *T. mesenterica*, plays a role in suppressing fungal host defenses. We identified a putative ascorbate oxidase in the MAGs of the lecanoromycete and *Tremella*, but not *Cyphobasidium*. When comparing six species of Tremellales with different trophic strategies, including the lichen-associated *Tremella* from this study and the three verified mycoparasites mentioned above, we found no clear trend in predicted secretome size, number of CAZymes and number of proteases. Likewise, the number enzymes potentially active on fungal cell walls (GH16-GH18, GH128, GH152) was similar regardless of ecology, and none could be shown to act exclusively on exogenous fungal polymers. Finally, N-auxotrophy of *Tremella* inferred from our data suggests *Tremella* has a biotrophic strategy, but our data do not allow us to speculate whether it retrieves nitrogen from one of the fungal partners, from the alga, or from other sources.

### Outlook

Our study is the first to provide complete genome assemblies for three fungal symbionts from metagenomic data. Until now, only one fungal symbiont has been assembled from whole lichen metagenomic DNA, the dominant lecanoromycete. Three innovations proved crucial. First, we employed warm water treatment of thalli to dislodge low coverage symbionts from the cortex EPS, thereby driving up coverage relative to the otherwise dominant lecanoromycete. Next, we employed recently developed algorithms to assign eukaryotic DNA to bins. Most previous lichen metagenomic studies (e.g., Greshake Tzovaras et al. 2020), relied on use of reference databases to bin their metagenomes. This allowed them to extract genomes similar to ones that already had been sequenced. Since no sequenced genome from the order Cyphobasidiales existed prior to our study, applying a reference-independent binning approach was crucial. Finally, we evaluated genome completeness based on phylogenetic relatedness. Taken together, these approaches open the door to direct assessment of multiple-eukaryote systems whilst bypassing the challenge of isolating and culturing individual members.

Our functional predictions for the three fungal genomes in the *Alectoria* lichen suggest that future experiments should focus on a possible role for yeasts in differential water retention through secretion of GXM-like polysaccharides as well as in P-scavenging, which previous studies suggest could be important in the oligotrophic conditions in which this lichen grows in nature (Johansson et al. 2011). Comparative studies combining assessment of yeast abundance with manipulation of wetting/drying cycles or provision of isotope-labeled nutrient precursors could be one way to answer these questions. Our predictions also suggest that more attention should be paid to the diverse pathogenicity factors secreted by the dominant fungus in the symbiosis, the lecanoromycete. RNA-Seq data may reveal whether these are upregulated in initial contact with algal symbionts or whether they could play a role in the aposymbiotic lifestyle of the fungus.

## Supplementary Material

Supplementary data are available at *Genome Biology and Evolution* online.

## Supplementary Material

evab047_Supplementary_Data
