# Erratum to: Predicted Input of Uncultured Fungal Symbionts to a Lichen Symbiosis from Metagenome-Assembled Genomes

**DOI:** 10.1093/gbe/evab129

**Published:** 2021-07-01

**Authors:** Gulnara Tagirdzhanova, Paul Saary, Jeffrey P Tingley, David Díaz-Escandón, D Wade Abbott, Robert D Finn, Toby Spribille

*Genome Biology and Evolution*, Volume 13, Issue 4, April 2021, evab047, https://doi.org/10.1093/gbe/evab047

In the originally published version of this manuscript incorrect supplementary data files were linked to the article. A correctionsnotice has been issued and correct supplementary files have now been uploaded..

