# Supplementary material for: Predicted Input of Uncultured Fungal Symbionts to a Lichen Symbiosis from Metagenome-Assembled Genomes
Source: Genome Biol Evol. 2021 Mar 9;13(4):evab047. doi: 10.1093/gbe/evab047 (PMC8355462; doi:10.1093/gbe/evab047)
Supplement: evab047_Supplementary_Data [file evab047_Supplementary_Data.zip › Tagirdzhanova_supplementary_figures_08022021.pdf]

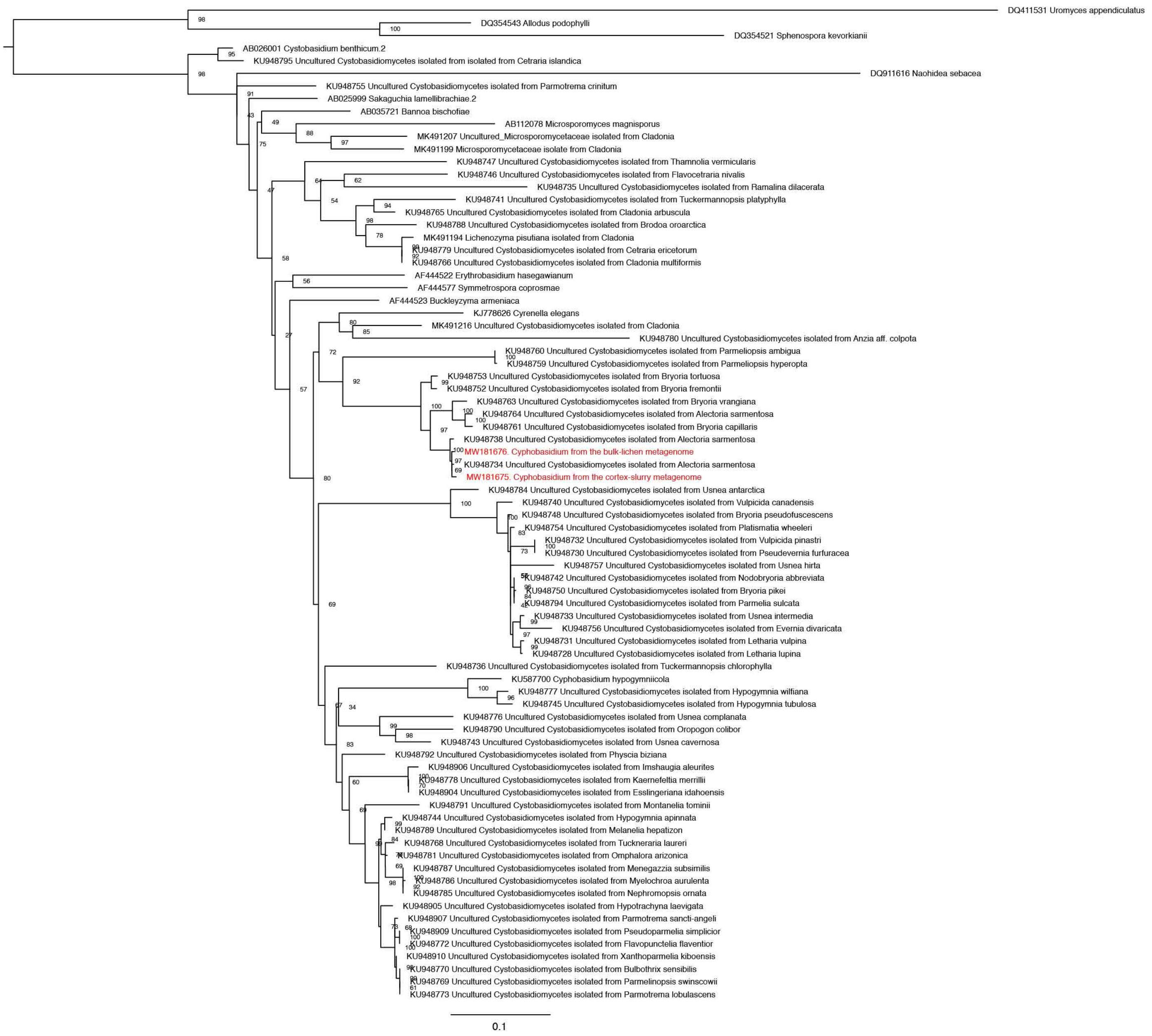

Fig. S1. Single-locus maximum likelihood tree of ITS rDNA for Cystobasidiomycetes. Branch lengths scaled to represent the expected number of substitutions per site. Bootstrap values are given by the nodes. Lineages retrieved from the studied metagenomes are indicated in red, the rest of the data is acquired from Genbank (Table S12)

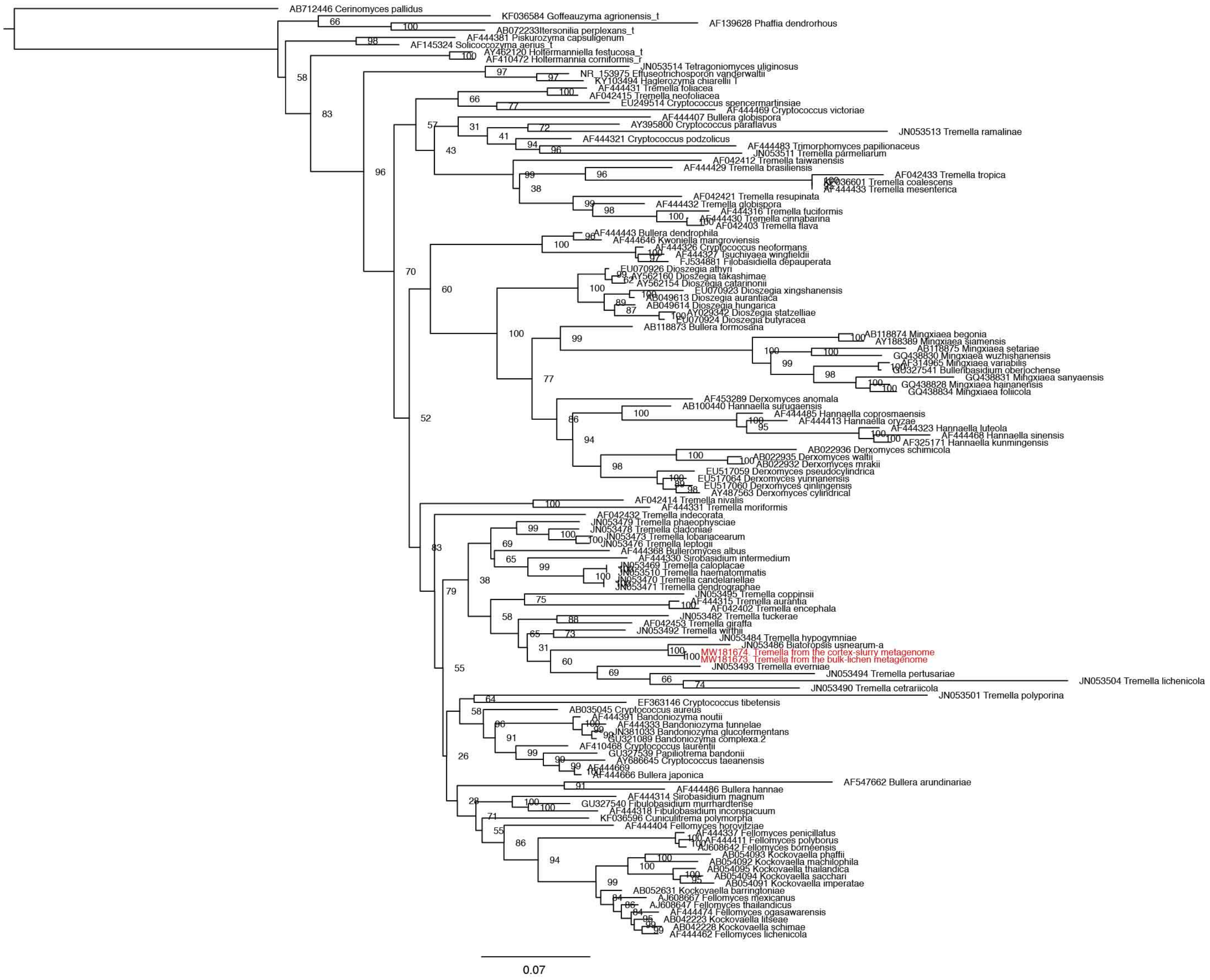

Fig. S2. Single-locus maximum likelihood tree of ITS rDNA for Tremellomycetes. Branch lengths scaled to represent the expected number of substitutions per site. Bootstrap values are given by the nodes. Lineages retrieved from the studied metagenomes are indicated in red, the rest of the data is acquired from Genbank (Table S12).

MAG	# proteins	# clusters	# singletons
Lecanoromycetes	9407	3453	4142
Cyphobasidium	6095	3365	2243
Tremella	6038	3275	2308

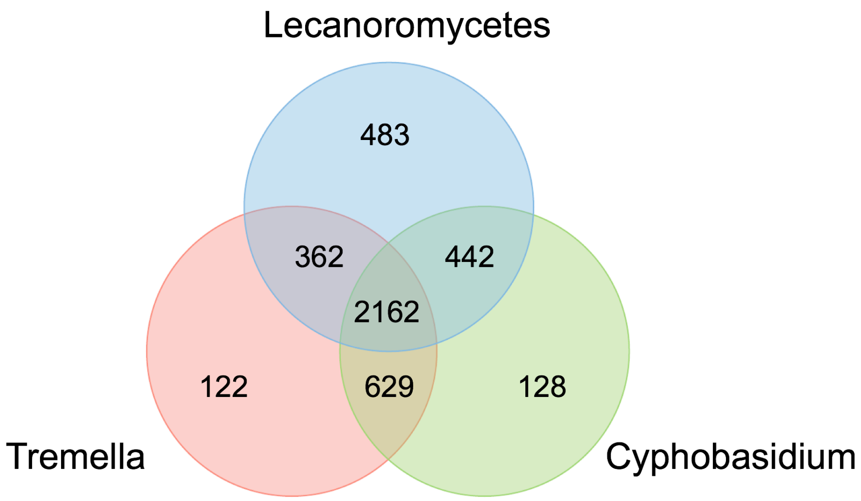

Fig. S3. Orthologous gene clusters shared by three fungal MAGs

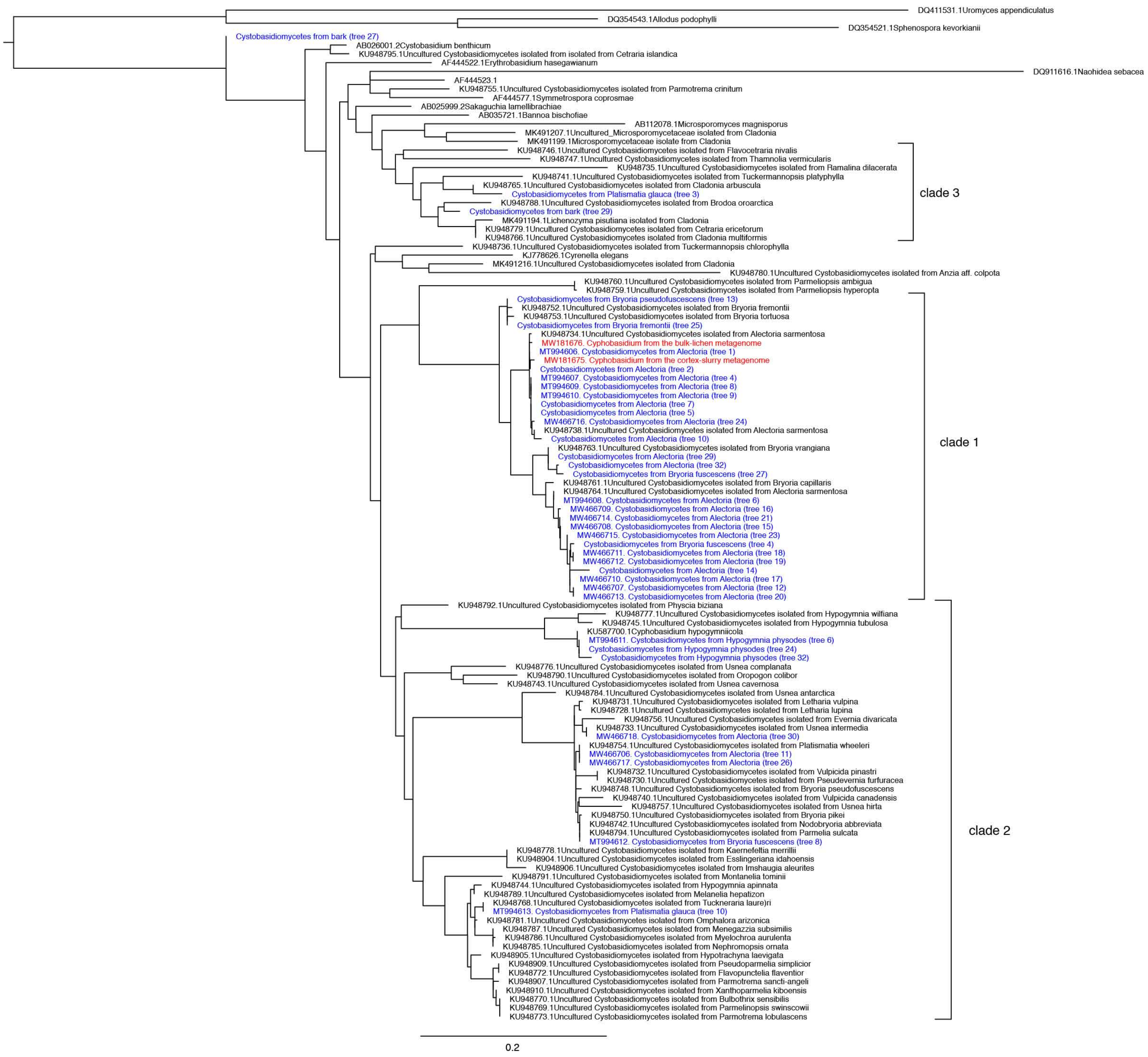

Fig. S4. Single-locus maximum likelihood tree of ITS rDNA for Cystobasidiomycetes. Branch lengths scaled to represent the expected number of substitutions per site. Sequences produced by PCR screening are indicated in blue, lineages retrieved from the studied metagenomes are indicated in red. For high quality sequences GenBank accession numbers are given, mid quality sequences were uploaded to Dryad repository (pending). The rest of the data is acquired from the Genbank (Table S12). Clades 1 to 3 correspond to clades shown in Figure 3.

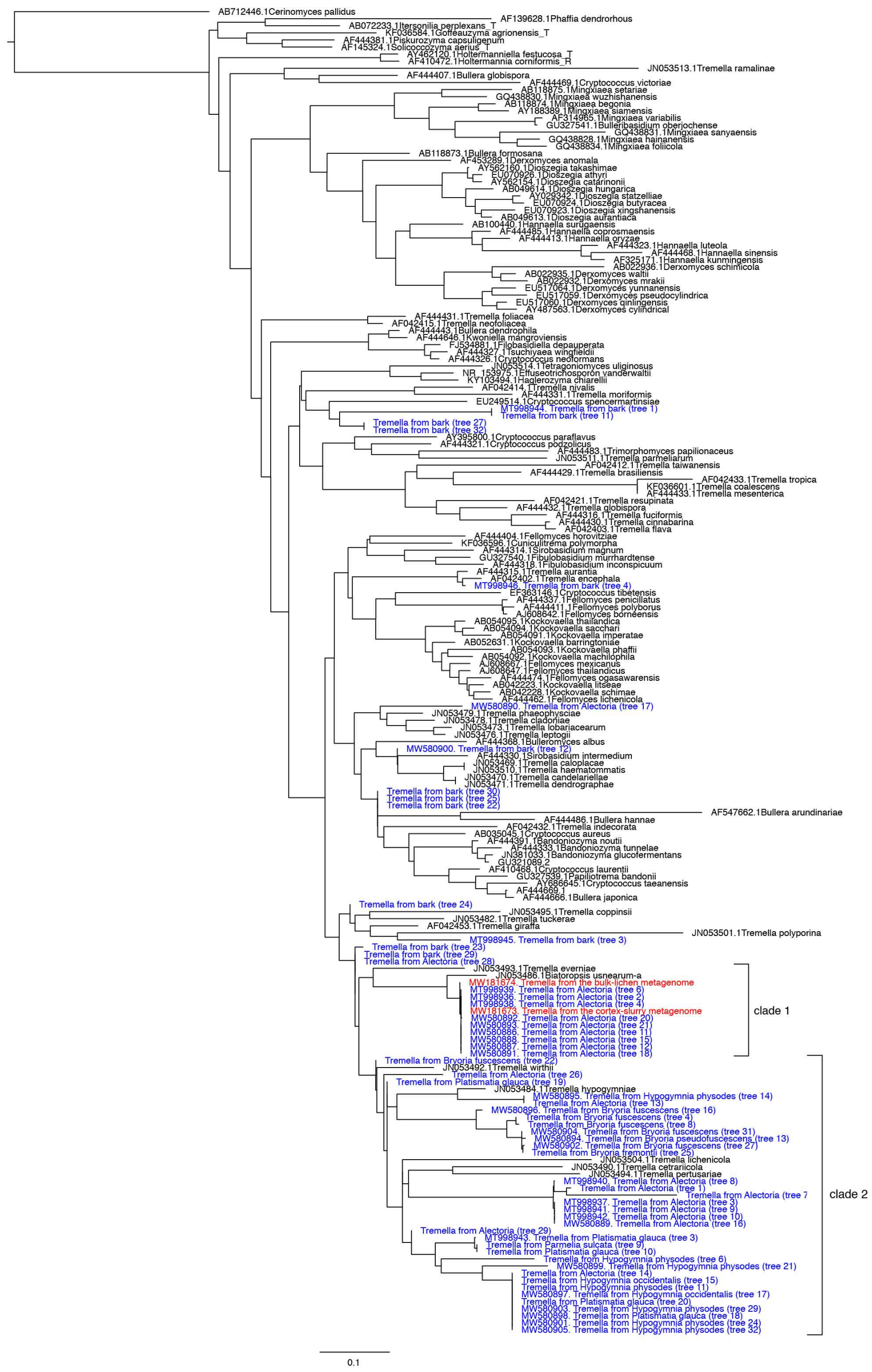

Fig. S5. Single-locus maximum likelihood tree of ITS rDNA for Tremellomycetes. Branch lengths scaled to represent the expected number of substitutions per site. Sequences produced by PCR screening are indicated in blue, lineages retrieved from the studied metagenomes are indicated in red. For high quality sequences GenBank accession numbers are given, mid quality sequences were uploaded to Dryad repository (pending). The rest of the data is acquired from Genbank (Table S12). Clades 1 and 2 correspond to clades shown in Figure 3.

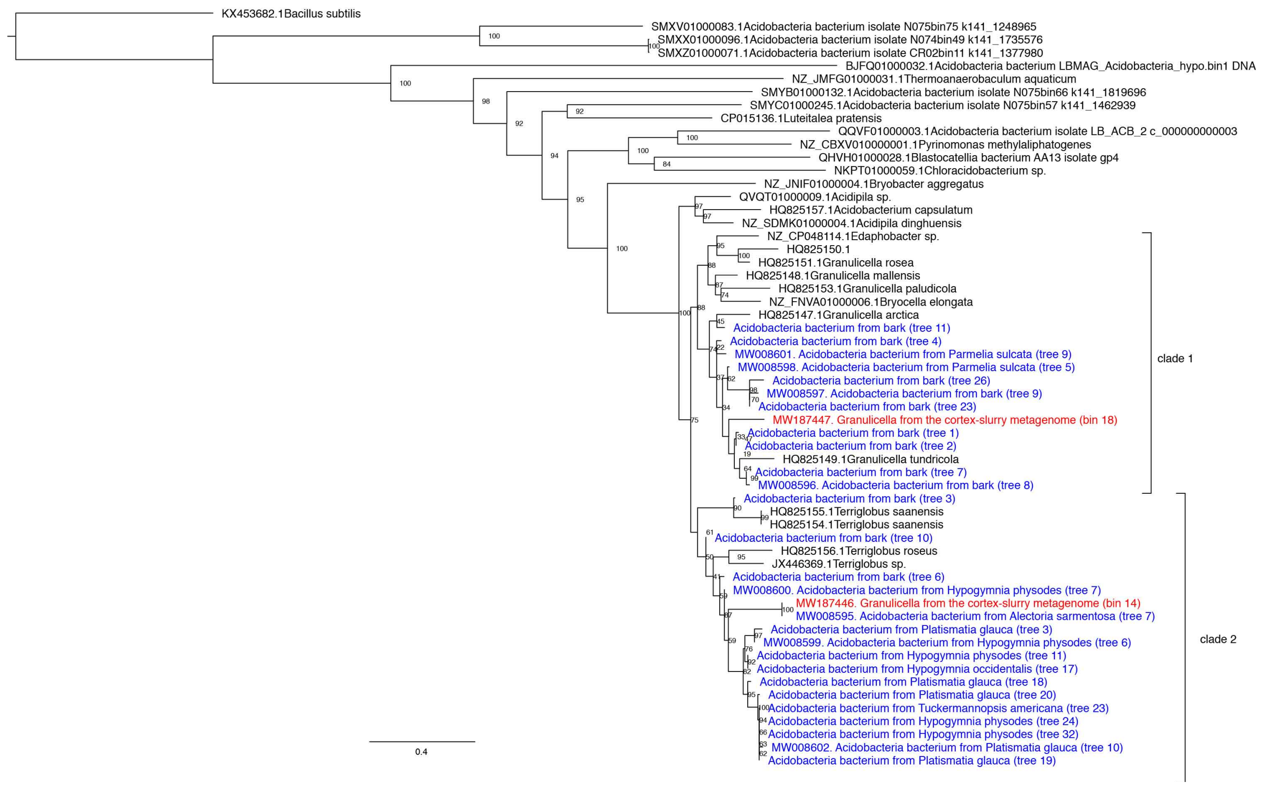

Fig. S6. Single-locus maximum likelihood tree of *rpo*B for Acidobacteria. Branch lengths scaled to represent the expected number of substitutions per site. Sequences produced by PCR screening are indicated in blue, lineages retrieved from the studied metagenomes are indicated in red. For high quality sequences GenBank accession numbers are given, mid quality sequences were uploaded to Dryad repository (pending). The rest of the data is acquired from Genbank (Table S15). Clades 1 and 2 correspond to clades shown in Figure 3.

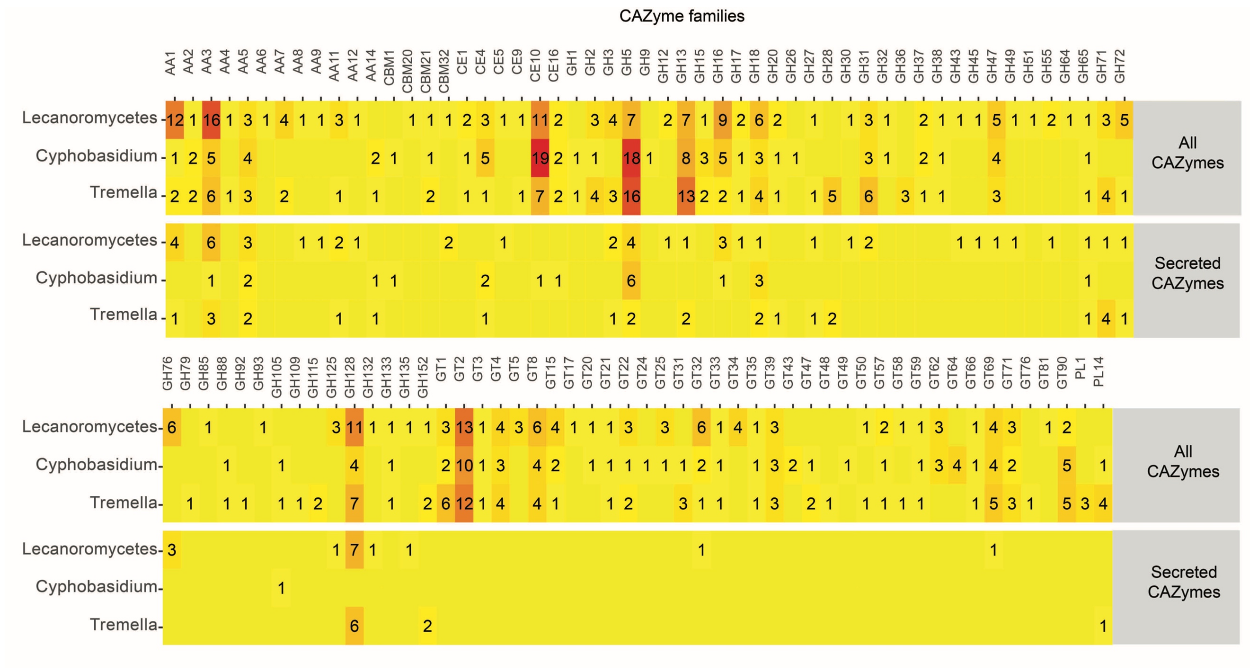

Fig. S7. Heatmap of CAZy families in three fungal MAGs; top panel shows all CAZymes; bottom panel shows only CAZymes predicted as secreted.

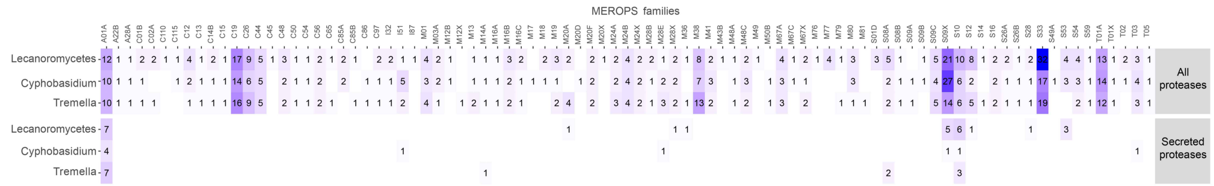

Fig. S8. Heatmap of MEROPS families in three fungal MAGs; top panel shows all proteases; bottom panel shows only proteases predicted as secreted.
